# KhufuEnv, an auxiliary toolkit for building computational pipelines for plant and animal breeding

**DOI:** 10.1186/s12859-026-06499-9

**Published:** 2026-06-03

**Authors:** Hallie C. Wright, Catherine E. M. Davis, Josh Clevenger, Walid Korani

**Affiliations:** 1 HudsonAlpha Institute for Biotechnology, 601 Genome Way Northwest, Huntsville, AL 35806 USA; 2 Veil Genomics, 601 Genome Way Northwest Suite 2021, Huntsville, AL 35806 USA

**Keywords:** Genomics, Auxiliary, Toolbox, Flexible, Breeding, Mapping, Pipelines, Data-processing

## Abstract

**Background:**

In the era of short- and long-read sequencing, vast amounts of DNA sequencing data are being generated. While a variety of tools exist for analyzing and manipulating genomics data, many have a finite number of functions, and thus, require users to depend on multiple sources for conducting analyses and processing data. An integrative environment of tools which is accessible to users of different computational backgrounds would facilitate more efficient data processing and level the playing field for researchers whose research depends on analyzing genomic data.

**Results:**

We developed the KhufuEnv, an open-source, auxiliary environment for manipulating and analyzing genomic and other datasets. As a proof of concept, we demonstrate rapid de novo identification of previously characterized quantitative trait loci (QTL) for cold tolerance in peanut and hairlessness in dog, identify a candidate sex-determination region (SDR) in *Amborella trichopoda* and calculate the proportion of the genome containing runs of homozygosity (ROH) in canine using previously published datasets.

**Conclusions:**

We introduce the KhufuEnv, which provides buildable tools for generating custom pipelines for analyzing a variety of genomic datasets from different species. The KhufuEnv is an open-source auxiliary environment available at https://github.com/w-korani/KhufuEnv. Its tools can be exploited for numerous applications and implemented for quick analysis supporting users with minimal computational experience.

**Supplementary Information:**

The online version contains supplementary material available at 10.1186/s12859-026-06499-9.

## Background

The emergence of DNA sequencing technology has brought forth a plethora of data. The National Center for Biotechnology Information (NCBI) Sequence Read Archive (SRA) contains over 36 petabytes (PB) of sequencing data to date [1]. In fact, genomic data is predicted to be the most demanding domain of data comparable to other big data generators like astronomy or YouTube [2]. Such data have revolutionized the capabilities of breeding programs and supported genomic selection [3] in animals [4] and plants [5]. The wealth of data being generated, however, demands the ability to analyze it in a high-throughput and efficient manner [6]. Thus, user-friendly tools which can support researchers with various computational skillsets are imperative for extracting relevant information from the masses of data being generated.

Several suites of tools have been developed for manipulating genomic data, including SeqKit [7, 8], Bioawk [9], Seqtk [10], SeqFu [11], VCFTools [12], BBTools [13], and BEDTools [14]. Some, such as VCFTools, Seqtk, and SeqFu, operate on limited data formats such as variant call format (VCF) files and FASTA/FASTQ files, respectively. Others, like Bioawk, focus primarily on parsing column and header information from genomic data files. Although these programs have immense value in their own respects, many of them require users to exploit additional software to produce final experimental results. We present the KhufuEnv, aimed to design a versatile and expansive environment for genomics data manipulation and trait mapping based on three principles: it requires simple processing, runs on a single thread, and can be completed quickly. To our knowledge, this is the largest collection of computational genomics tools within a single environment to date.

**Fig. 1:**
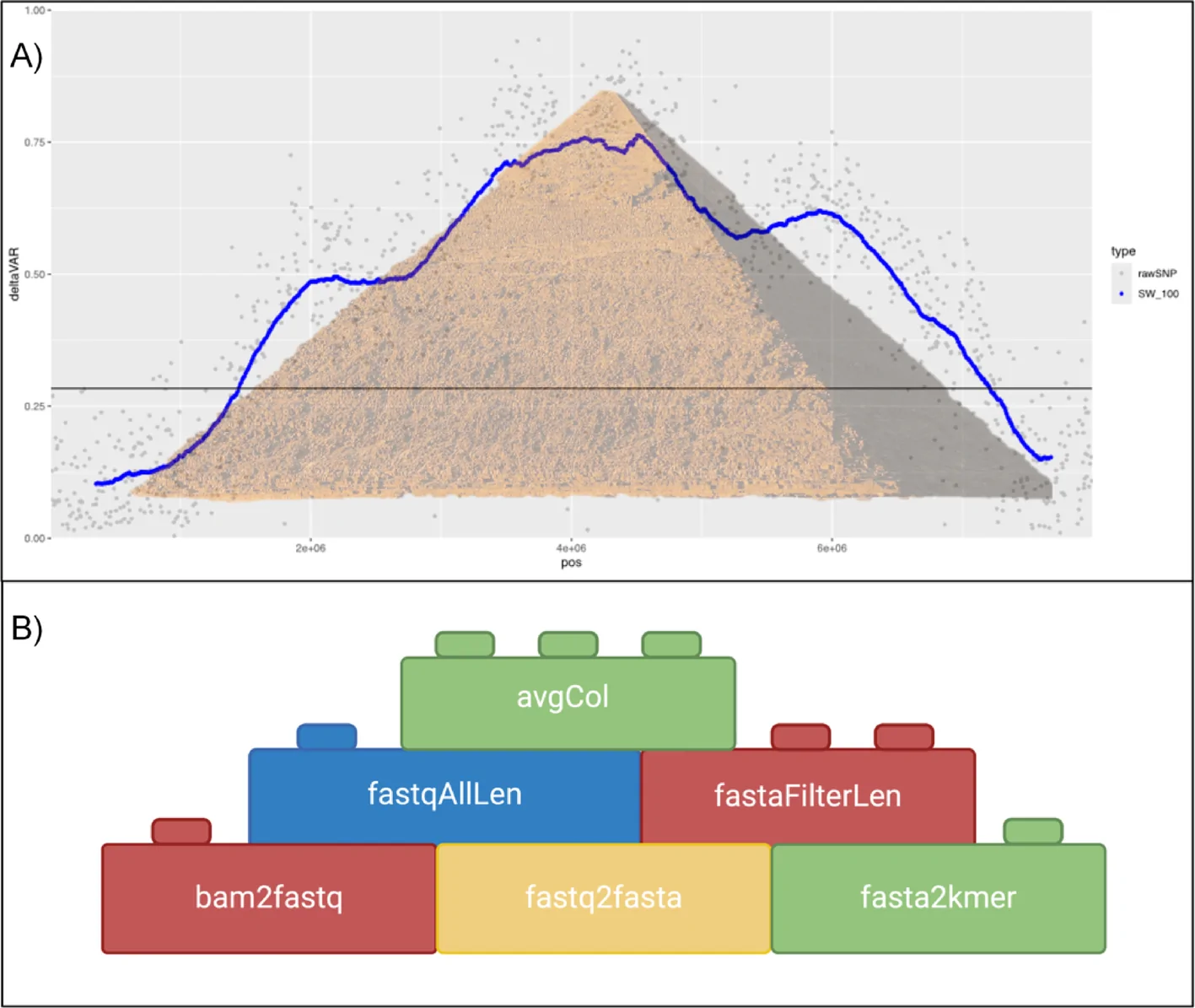
**A** Inspired by the visualization of QTL mapping peaks and subsequently named after the pyramid of Khufu, the KhufuEnv is an extension of the Khufu platform described by Korani et al. 2021. **B** The KhufuEnv is an auxiliary environment containing modular tools that facilitate building customized pipelines

Visually similar to the shape of a quantitative trait locus (QTL) peak (Fig. 1A), the Khufu platform (https://www.hudsonalpha.org/khufudata/), a now full-service genotyping platform at HudsonAlpha Institute of Biotechnology, was named after the Great Pyramid of Khufu [15]. In their 2021 study, Korani et al. [16] demonstrated Khufu’s utility for conducting QTL-mapping with low-coverage sequencing of whole populations to de novo identify QTL for blanchability, which were validated in an independent population. This same platform also confirmed de novo QTL identified in previously described datasets [17, 18]. Although Khufu was originally conceived for mapping traits in complex polyploids like peanut (*Arachis hypogaea* L.), the KhufuEnv, an extension of the Khufu platform, contains versatile tools which can be exploited across a variety of plant, animal, human, and microbial systems. The KhufuEnv encompasses tools that not only assist with QTL mapping but processing of genomic data and other file types.

The KhufuEnv is an auxiliary environment containing standalone and modular tools which support custom analyses of high-throughput sequencing data (Fig. 1B). Here, we summarize the core sections of the KhufuEnv, introduce our custom HapMap and PanMap formats, and detail four case studies demonstrating the efficiency and utility of the tools in this environment.

## Implementation

The KhufuEnv was designed to support genomic analyses from simple tasks to buildable pipelines. Written in Bash and C+ + (implemented through Rcpp) and leveraging R [19] (data.table, tidyr, and plyr libraries) and gawk, the KhufuEnv contains over 100 single-threaded functions that require minimal computational resources and can be run interactively or in a shell script. The functions are housed in a simple Bash script that is sourced after following installation instructions on https://github.com/w-korani/KhufuEnv. Briefly, the environment can be cloned from GitHub using the command line. The installation script is then run from the KhufuEnv folder, and the software is integrated into the user’s .bashrc. The installer script checks for required R packages and automatically installs any that are absent. The KhufuEnv can be uninstalled by running the uninstaller script from the KhufuEnv folder and removing the source command from the user's .bashrc. As a Bash-based tool, the KhufuEnv requires a Unix-like terminal environment and is compatible with Linux, macOS, and Windows systems with Bash emulation.

The KhufuEnv contains six sections with 132 tools for manipulating genomics files and building custom pipelines, housed in separate Bash scripts that are sourced from the environment. The sections include: KhufuEnvHapMap, KhufuEnvPanMap, KhufuEnvStats, KhufuEnvFASTA, KhufuEnvFASTQ, and KhufuEnvDataset. The following overview is not exhaustive of all tools. After one loads the KhufuEnv, the command *KhufuEnvHelp* can be executed to list all tools. More detail regarding individual tools can be retrieved by running the command *KhufuEnvHelp [tool name]*. For instance, to learn more about how to use the tool vcf2hapmap, one could run *KhufuEnvHelp vcf2hapmap* and obtain information regarding the tool’s description, parameters, and example commands associated with practice files located in the KhufuEnv (Supplementary Fig. 1).

### KhufuEnvHapMap

Variant calls are housed in a variety of file formats. Two popular formats are VCF [12] and HapMap [20] files. In the KhufuEnv, we have created a modified version of the “standard” HapMap (Supplementary Fig. 2) which contains the most essential information regarding variant calls (Supplementary Fig. 3). A HapMap can easily be converted to a Khufu HapMap with the stdhapmap2hapmap command. The Khufu HapMap includes columns that correspond to chromosome, position, and sample names for genotyping calls at their respective sites. The Khufu HapMap has dropped columns for reference SNP identifier (rs#), alleles, strand, reference assembly version (assembly#), genotyping center (center), protocol identifier (protLSID), genotyping assay identifier (assayLSID), panel of individuals genotyped (panelLSID), and quality control for entries (QCcode). Such information were perhaps more relevant to human studies conducted within the International HapMap Project [21] yet are trivial to the actual calling of variants. Allele calls with the value “NA” are converted to “–,” and homozygous allele calls are condensed to a single allele; i.e., “AA” is converted to “A”. Heterozygous allele calls still maintain information for two alleles. Henceforth in this paper, HapMap will refer to the Khufu HapMap format.

The KhufuEnvHapMap section contains several tools for processing HapMap files. Genotyping files can be easily converted between text, VCF, standard HapMap, and HapMap. Note that values for filter, quality, and depth of coverage will not be present when converting HapMap to VCF since this information is not included in HapMap format. Often in genomic analyses, calls must be filtered to achieve a set of high confidence variants by removing potentially false variants. Within the KhufuEnv, HapMap variants can be filtered for those which are diallelic, single nucleotide polymorphisms (SNPs), or structural variants (SVs) and meet specified missing data and minor allele frequency (MAF) criteria. Similarly, the KhufuEnv allows for filtering out samples which have a percentage of missing variant calls.

### KhufuEnvPanMap

**Fig. 2:**
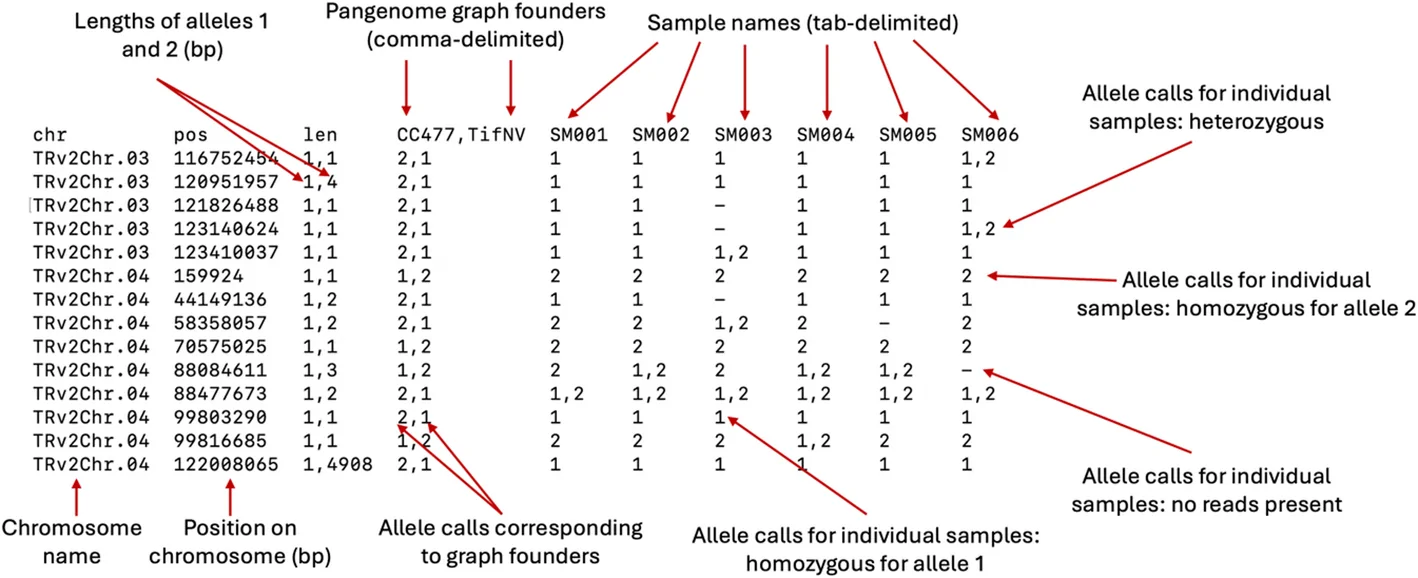
The Khufu PanMap file format concisely displays variants against a pangenome graph, outlining both short and long variants side-by-side. Arrows designate descriptions for different parts of the file. If a pangraph member has a genotyping call of “0,” it indicates that no data covers the variant. If a genotyped sample has a call of "-," it indicates that no data covers the variant

Visualizing long and short variants side-by-side from pangraph-generated calls can be a challenge using traditional file formats. For instance, VCFs may display large strings of DNA to represent a long SV, which is challenging and often causes complications for visualizing alleles on a command line or external (e.g. Excel) interface. We resolve such issues with the development of our custom PanMap format (Fig. 2). The PanMap format is similar to the HapMap format in that we retain information regarding chromosome, position, and calls for samples. However, the PanMap format also includes columns that list the length of variants called across parents and the allele call for each parent (designated numerically). Sample allele calls are encoded numerically with respect to their parental allele’s numerical code, and heterozygous sample calls include a comma to separate the alleles. Like the KhufuEnvHapMap section, there are several tools for processing PanMap files. PanMaps can be converted to and from HapMap, VCF, and heatmaps, and PanMaps can be converted to text. PanMaps can also be filtered according to user-specified thresholds for sample-level missing data, variant-level missing data, and MAF. While the PanMap was named according to its utility in housing genotyping calls from pangraph graphical fragment assembly (GFA) references, the PanMap file type can also be applied to calls generated from a linear reference using the hapmap2panmap or vcf2panmap tools.

### KhufuEnvStats

The KhufuEnvStats section covers tools that allow for easy extraction of statistics including average, median, standard deviation, sum, and minimum and maximum values for factors, columns, or rows in a dataset.

### KhufuEnvFASTA

This section contains tools to facilitate converting FASTAs to text files with fasta2txt and vice-versa with txt2fasta. Files can be split into individual chromosomes with fastaSplit, and sequences within a single file that have the same header can be combined in the same file with fastaCombine. Sequence length for each chromosome can be extracted with fastaSeqLen, and the entire sequence length can be extracted with fastaLen. FASTA files can be artificially k-merized with fasta2kmer, specifying window width and step size.

### KhufuEnvFASTQ

Like some of the tools in the KhufuEnvFASTA section, the KhufuEnvFASTQ section allows for manipulating FASTQ files which can aid in preprocessing DNA sequence data prior to analysis. FASTQs can readily be converted to FASTA and vice-versa with the fastq2fasta and fasta2fastq tools. The entire length of nucleotides can be extracted with fastqAllLen, which is useful for calculating depth of coverage without prior knowledge of the sequencing depth for FASTQ files. The fastqLen tool can be used to print the length of each read individually from a FASTQ file, and the fastqAverage tool can be used to print the average length of reads. FASTQs can be filtered by minimum read length with the fastqFilterLen tool, subsampled for a desired total nucleotide length with fastqSubSampling, and artificially k-merized with fastq2kmer. The phred33 and phred64 tools print quality score information for the user’s reference.

### KhufuEnvDataset

The KhufuEnv is not exclusive for facilitating analysis of genomics data but can be extended to several other data types in the KhufuEnvDataset section. Similar tools to those presented in the HapMap and PanMap sections, merge, subtract, and combineSimilarColumnDS can be applied to other datasets here. This section also includes tools which may be relevant to preparing datasets for statistical analyses such as long2wideDS and wide2longDS and extracting metadata for datasets with tools like dimDS, maxLenDS, and minLenDS.

## Results

To demonstrate the utility of KhufuEnv for analyzing diverse datasets and developing customized genomics pipelines, we outline four case studies using publicly available data and benchmark its tools against similar functions in known genomics toolboxes. Detailed methods for the case study examples and benchmarking can be found in Supplementary File 1 and Supplementary Code 1.

### Case study (1) Confirming a candidate locus for cold tolerance in peanut (*Arachis hypogaea* L.)

**Fig. 3:**
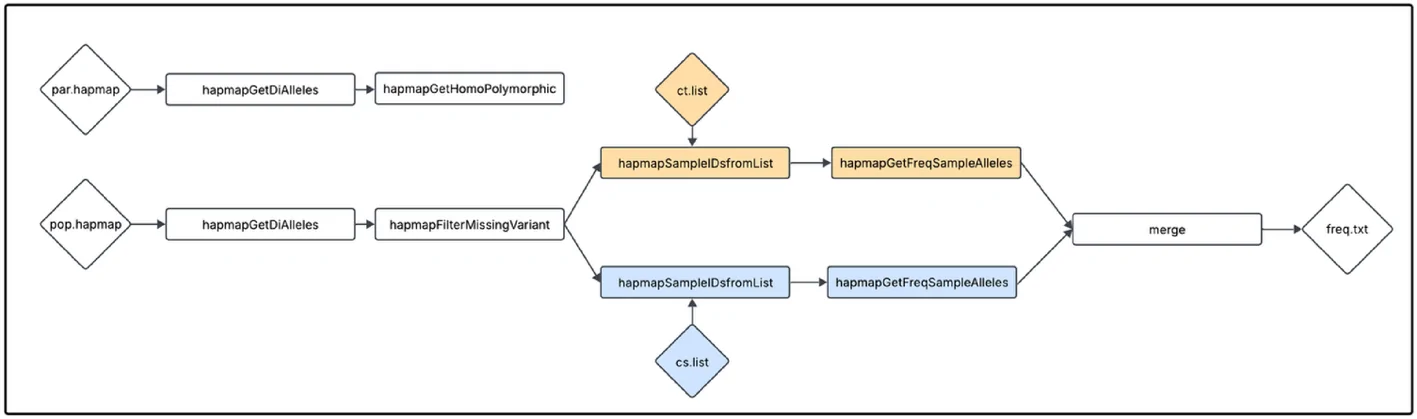
The custom KhufuEnv pipeline used for identifying a cold tolerance QTL in peanut

Peanut is a globally important oilseed and food crop. While it thrives in tropical and subtropical regions, it is cultivated in over 100 countries under a variety of agroecological environments [22]. The increasing demand for peanut [23] requires the expansion of production to high-latitude areas [24]. However, cold temperatures negatively impact peanut development [24] and can be particularly detrimental to germination [25]. To aid marker-assisted breeding for chilling tolerance in peanut, Zhang et al. [26] identified a QTL for cold tolerance during germination in a recombinant inbred line (RIL) population from a bi-parental cross of a cold-susceptible (DF12) and cold-tolerant (Huayu 44) parent. The cold-susceptible and cold-tolerant parents were sequenced to a depth of coverage of 22.27 × and 18.7x, respectively, and 200 random RILs were sequenced to an average depth of coverage of 5.76x (NCBI SRA accession number PRJNA931845). We exploited the KhufuEnv to demonstrate the rapid de novo identification of this QTL using the dataset by Zhang et al. [26] (Fig. 3).

A parental HapMap for the cold-susceptible (DF12) and cold-tolerant (Huayu 44) parents was constructed by using the Khufu pipeline described by Korani et al. [16] (https://www.hudsonalpha.org/khufudata/) which uses BWA [27] to align reads from both samples to the TifRunner version 2 peanut assembly (https://data.legumeinfo.org/Arachis/hypogaea/genomes/Tifrunner.gnm2.J5K5/) and generates variant calls with a minimum read depth per variant of five. The hapmapSNP2SV tool was used to reformat the HapMap to distinguish SVs from SNPs with commas. The variants in the parental HapMap were then filtered to retain those which were diallelic with hapmapGetDiAlleles and homopolymorphic with hapmapGetHomoPolymorphic. A text file containing relative germination rates from Zhang et al. [26] for each line across five independent experiments was used to extract 20 samples with the highest germination rates as cold-tolerant (ct) and the 20 samples with the lowest germination rates as cold-susceptible (cs). The sample names were used to generate ct and cs list files.

**Fig. 4:**
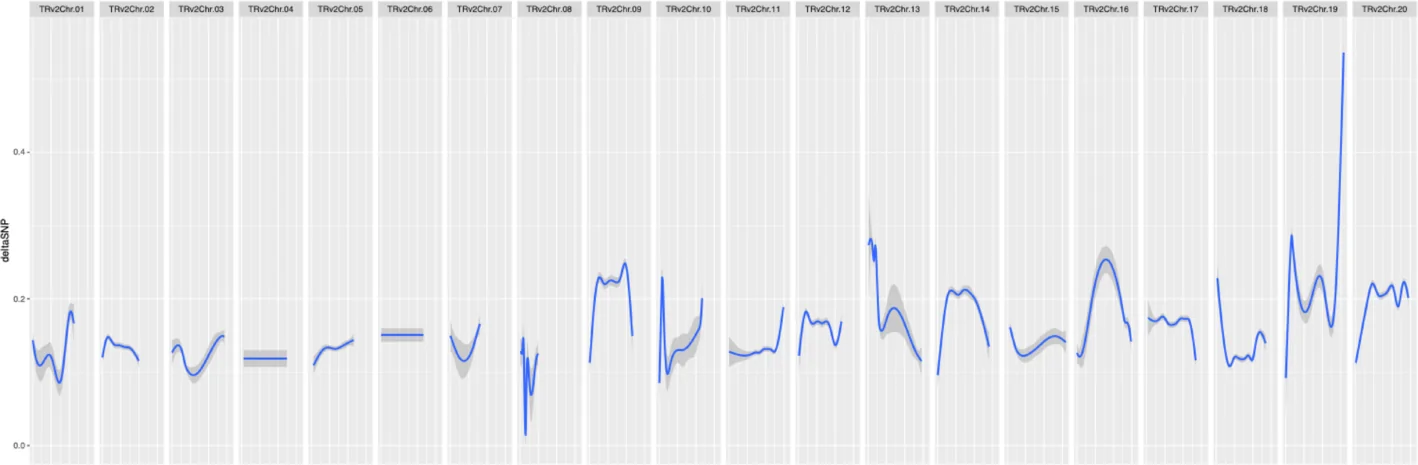
Tools in the KhufuEnv enable the calculation for the ΔSNP-index for 20 ct and 20 cs samples across the peanut genome, which demonstrate a strong association near 155.2 Mb on Chr19 for cold tolerance at germination

A population HapMap was generated by using the Khufu pipeline as described above with a minimum read depth per variant of three. The population HapMap was filtered to include only diallelic variants with hapmapGetDiAlleles and remove variants with > 50% missing data with hapmapFilterMissingVariant. Using two text files which contain the names of cs and ct samples, the population HapMap was split into separate cs and ct HapMaps with hapmapSampleIDsfromList. The hapmapGetFreqSampleAlleles tool was used to obtain allele frequencies, and each of the frequencies for the cs and ct samples were combined into a single text file using the merge tool. This allele frequency text file was processed to calculate ΔSNP-index, i.e., the difference in allele frequencies across the two populations, and extract candidate regions which exhibited the highest ΔSNP-index across all environments. The highest ΔSNP-index is located near 155.2 Mb on chromosome 19 (B09) (Fig. 4 and Supplementary File 2), located within the same region that Zhang et al. [26] fine-mapped the QTL to.

### Case study (2) Confirming a sex-determination region (SDR) previously identified in *Amborella trichopoda*

The transition from monoecy, the presence of male and female organs on the same plant, to dioecy, the presence of male and female organs on separate plants, has independently evolved hundreds of times in flowering plants [28]. Dioecy is estimated to occur in 5–10% of flowering plants and is the result of genetically controlled sex determination systems [28]. SDRs are genomic regions which rarely recombine and contain sex-determining genes [29]. Characterizing sex chromosomes and SDRs in plants is relevant for understanding genome evolution and has implications for plant breeding, particularly for fruiting crops [30].

**Fig. 5:**
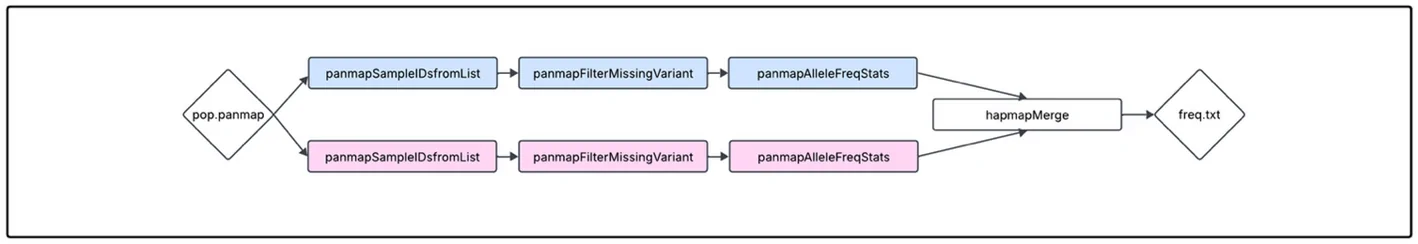
The custom KhufuEnv pipeline used for confirming previously identified SDR in *Amborella*

As a sister to all extant flowering plants [31] and a dioecious plant [32], *Amborella trichopoda* is an ideal system to study sex chromosome evolution in flowering plants [31, 33]. Recent studies have shed light on its ZW sex determination system [31], but the genes involved in dioecy in *Amborella* have previously remained unknown. Carey et al. [34], however, recently characterized the SDR and identified candidate genes for sex determination in *Amborella*. We use the whole-genome sequencing (WGS) data for 104 *Amborella* samples (NCBI SRA accession number PRJNA1161132) and the Santa Cruz 75 version 2 assembly published by Carey et al. [34] to demonstrate the KhufuEnv capabilities in identifying trait-associated regions in allosomes (Fig. 5).

**Fig. 6:**
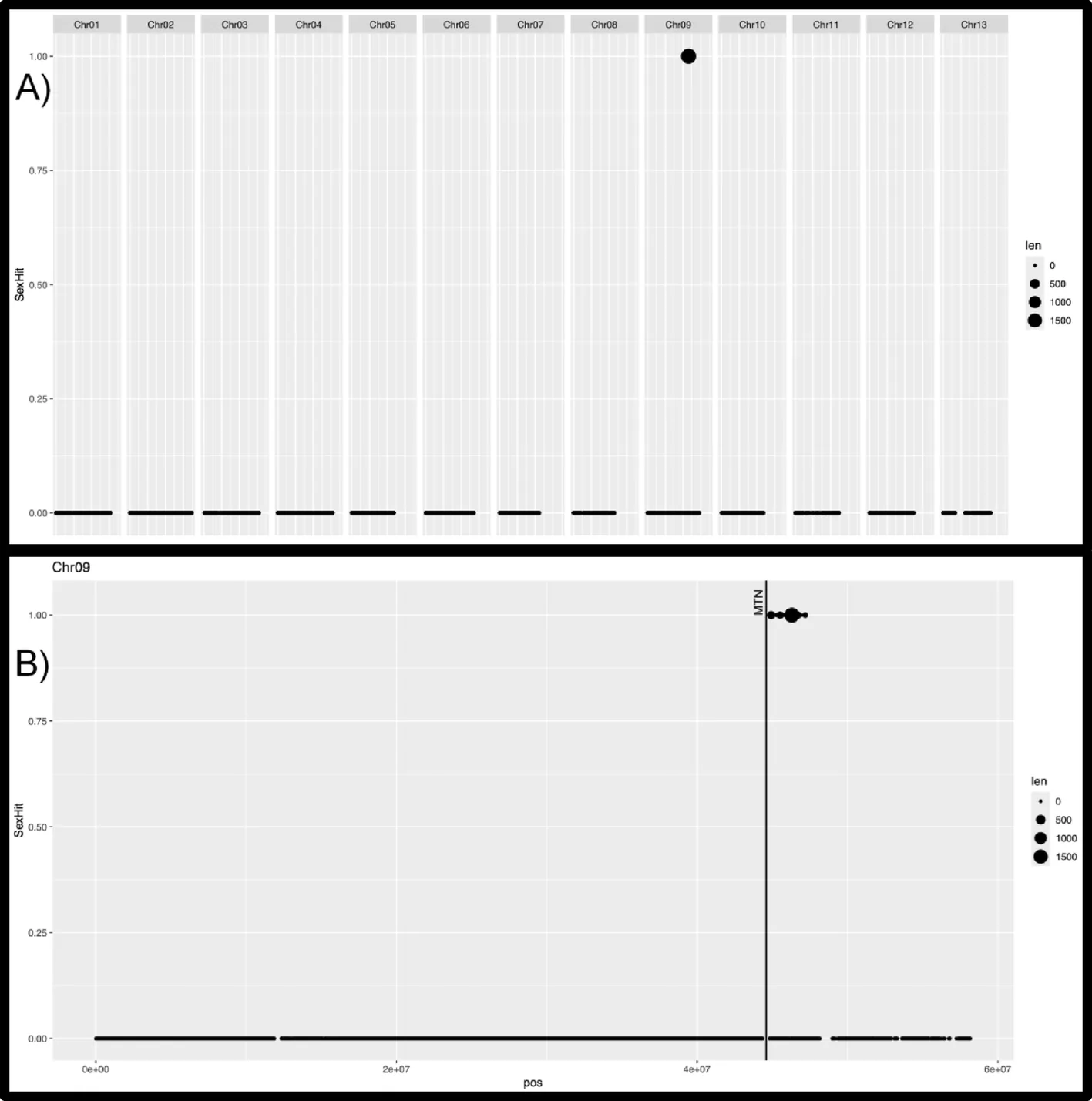
**A** A single SDR on chromosome 9 contained allele frequencies which differed by > 0.75 between male and female WGS samples. **B** The SDR identified with a custom Khufu pipeline was 246 kb downstream to *MTN1-2*, a candidate sex-determination gene identified Carey et al. [34]. “Len” represents the maximum length of an allele variant at a respective location

Briefly, the KhufuPAN pipeline [35] (https://github.com/w-korani/KhufuPAN) was implemented to generate a pangenome for HAP1 and HAP2 of the Santa Cruz 75 assembly in the form of a GFA file with Minigraph-Cactus [36]. After retaining high-quality variants, Giraffe [37] was used for mapping reads from the *Amborella* samples to the pangenome. A variant file in the form of a PanMap was generated using a minimum depth filter of two reads per variant. Initially, two list files were generated corresponding to male and female individuals described by Carey et al. [34] and panmapSampleIDsfromList was used to generate separate male and female PanMaps. Each PanMap was filtered to remove variants with data missing from > 75% samples with panmapFilterMissingVariant. Allele frequencies were obtained for each PanMap with panmapAlleleFreqStats. The column information for the chromosome, position, variant length, and parental calls from the PanMap was added to the allele frequencies to generate female and male allele frequency statistics files. The male and female allele frequency statistics files were then merged using hapmapMerge. The merged file was manually filtered to remove redundant parental call and length columns and columns containing allele count and sum. Variants with allele frequencies which differ by > 0.75 were mined for putative candidate regions for SDR (Supplementary File 3). A single region between 44,825,527–47,193,113 bp on chromosome 9 contained allele frequencies which differed by 1.0 (Fig. 6A). This region was 245,680 bp downstream to the *MTN1-2* gene Carey et al. [34] identified as a candidate sex-determining gene (Fig. 6B).

### Case study (3) Estimating runs of homozygosity (ROH) in canine

**Fig. 7:**
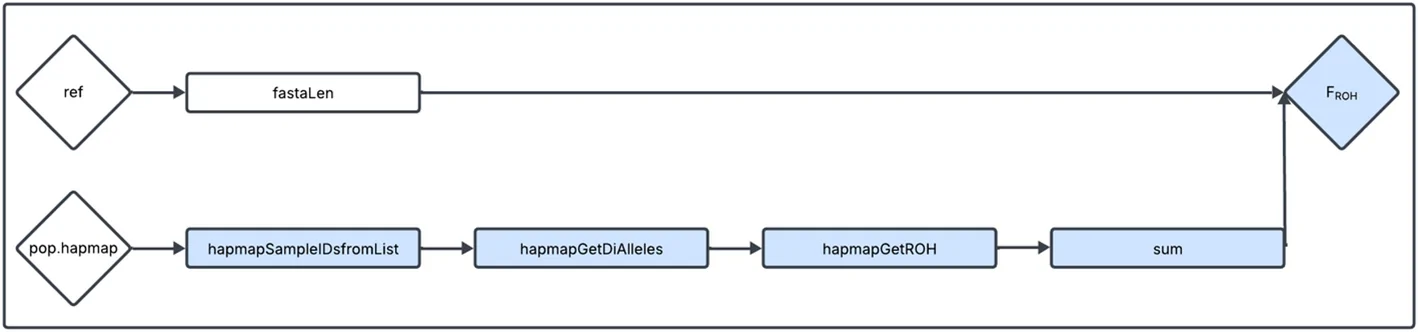
The custom KhufuEnv pipeline used for calculating ${\mathrm{F}}_{\mathrm{R}\mathrm{O}\mathrm{H}}$ in canine

Runs of homozygosity (ROH) [38] are long stretches of homozygous regions resulting from the inheritance of two copies of an ancestral haplotype. ROH can be used in animal breeding as measure of inbreeding within individuals or populations [39], and consequently, can be an indicator of selection [40, 41]. In canines, ROH varies greatly across and within breeds in part due to inbreeding associated with selection [42, 43]. Greater ROH may be particularly alarming given that stretches of homozygosity often harbor deleterious alleles associated with disease [43] and may be an artifact of recent canine inbreeding [41]. Meadows et al. [44] conducted short-read sequencing on nearly 2,000 candid samples at 20 × depth of coverage to assess how ROH varies across over 300 dog breeds, village dogs, wolves, and coyotes. While Meadows et al. [44] and others [45–49] calculate ROH using PLINK [50], we present a method for quickly calculating ROH and fraction of ROH (${F}_{ROH}$) in the KhufuEnv (Fig. 7).

**Fig. 8:**
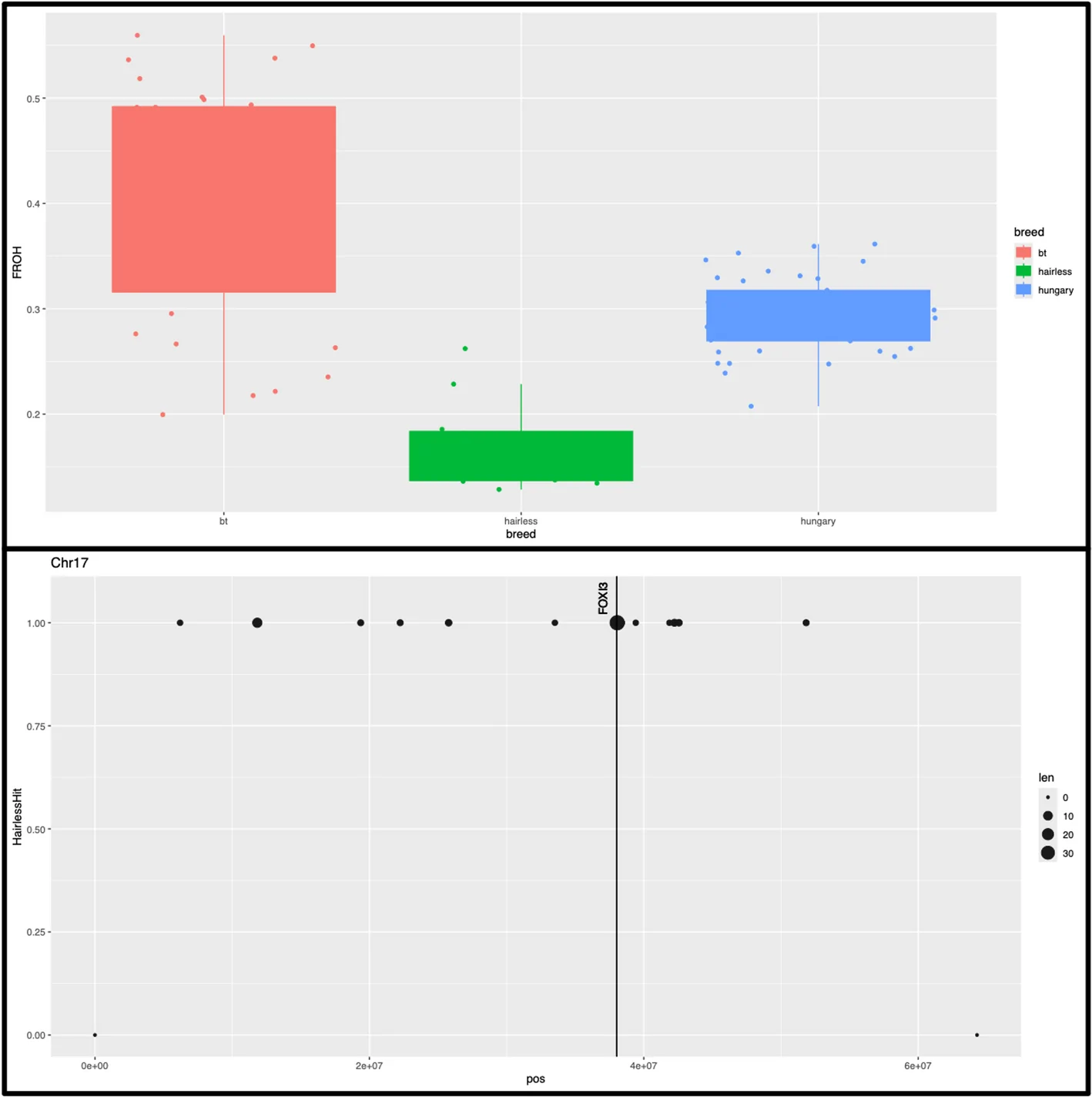
${\mathrm{F}}_{\mathrm{R}\mathrm{O}\mathrm{H}}$ for three canine breeds using tools in the KhufuEnv. As was reported by Meadows et al. [44], hairless dogs have relatively low ${\mathrm{F}}_{\mathrm{R}\mathrm{O}\mathrm{H}}$ while bt dogs have relatively higher ${\mathrm{F}}_{\mathrm{R}\mathrm{O}\mathrm{H}}$ and Hungary dogs have ${\mathrm{F}}_{\mathrm{R}\mathrm{O}\mathrm{H}}$ in between

To calculate ${F}_{ROH}$, we first used the fastaLen tool to determine the total sequence length of the German Shepherd reference genome [51] (UU_Cfam_GSD_1.0, GCF_011100685.1). The variant calls published by Meadows et al. [44] in the form of a VCF (https://www.ebi.ac.uk/ena/browser/view/PRJEB62420) were converted to a HapMap using vcf2hapmap. The HapMap was filtered to keep only variants that were diallelic and was then split by breed using hapmapSampleIDsfromList. The hapmapGetROH tool was then used to extract regions of ROH per sample. ROH lengths were added together for each sample using sumCol, and the sums of ROH per sample were divided by the genome size to determine ${F}_{ROH}$. ${F}_{ROH}$ values for each breed’s HapMap (Supplementary File 4) were plotted in R (Fig. 8). These aligned with the observations by Meadows et al. [44].

### Case study (4) Mapping QTL associated with hairlessness in canine

**Fig. 9:**
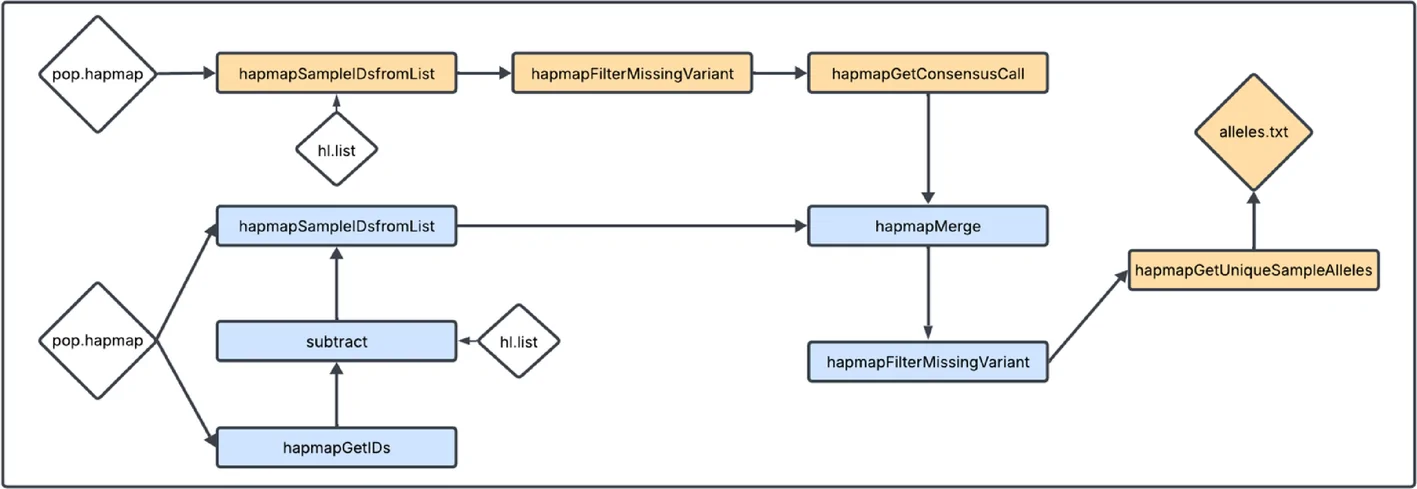
The custom KhufuEnv pipeline used for identifying QTL for CED in canine

Canine ectodermal dysplasia (CED) is a condition commonly found in Mexican and Peruvian hairless dogs and Chinese crested dogs which results in hairlessness and abnormal tooth morphology [52, 53]. CED is a semidominant, autosomal trait [54] for which heterozygous individuals exhibit the hairless phenotype while homozygotes are lethal. Previously, CED has been mapped to a region on chromosome 17 [52, 55], and further mutation and expression analyses supported the identification of the transcription factor *FOXI3* as a candidate gene for ectodermal development [52]. Specifically, the mutant allele of *FOXI3* harbors a frameshift mutation likely rendering the protein nonfunctional [52]. Using the genotyping data published by Meadows et al. [44] across 1600 dog samples including those which vary for the hairless trait, we attempted to recapitulate the candidate region associated with CED identified by Drögemüller et al. [52] using the KhufuEnv tools (Fig. 9).

**Fig. 10:**
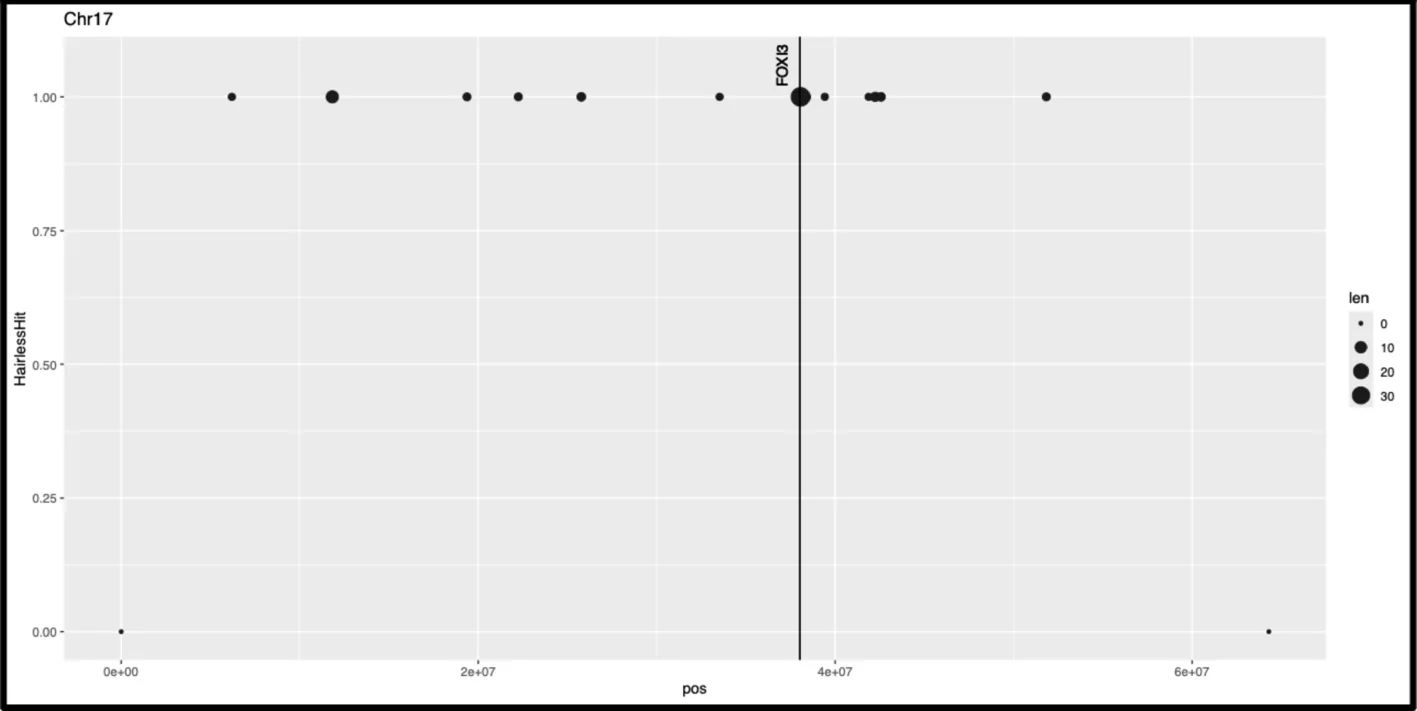
Identifying unique sample alleles between hairless and non-hairless dogs in the KhufuEnv identified a candidate region harboring a gene previously characterized by Drögemüller et al. [52] for CED in canine. “Len” represents the maximum length of an allele variant at a respective location

The previously generated HapMap for the variant calls by Meadows et al. [44] was used to extract breed names with hapmapGetIDs. A list of non-hairless dog breeds was constructed by using the subtract tool to remove a list of the 11 hairless breeds from the initial sample list. Due to its large size, the initial VCF was split into 165 subsets (subsets of 100,000 variants) to allow for faster processing. The VCFs were converted to HapMaps with the vcf2hapmap tool. HapMaps corresponding to the hairless samples were generated with the hapmapSampleIDsfromList tool, supplied by the list of 11 hairless breeds. Variants were filtered to remove those with > 50% missing data, and the hapmapGetConsensusCall tool under default settings was used to extract the most common allele for each of the variants in the hairless samples. Then, HapMaps corresponding to the non-hairless samples were generated with the hapmapSampleIDsfromList tool using the non-hairless samples list. The non-hairless HapMaps were merged with the consensus hairless HapMap using hapmapMerge, and the merged HapMap was filtered to remove variants with > 50% missing data with hapmapFilterMissingVariant. Alleles unique to the hairless samples were extracted using hapmapGetUniqueSampleAlleles, and these were mined for candidate regions associated with CED. One region corresponded with the previously identified *FOXI3* gene associated with CED (Fig. 10 and Supplementary File 5).

### KhufuEnv tools are flexible without compromising efficiency

**Fig. 11:**
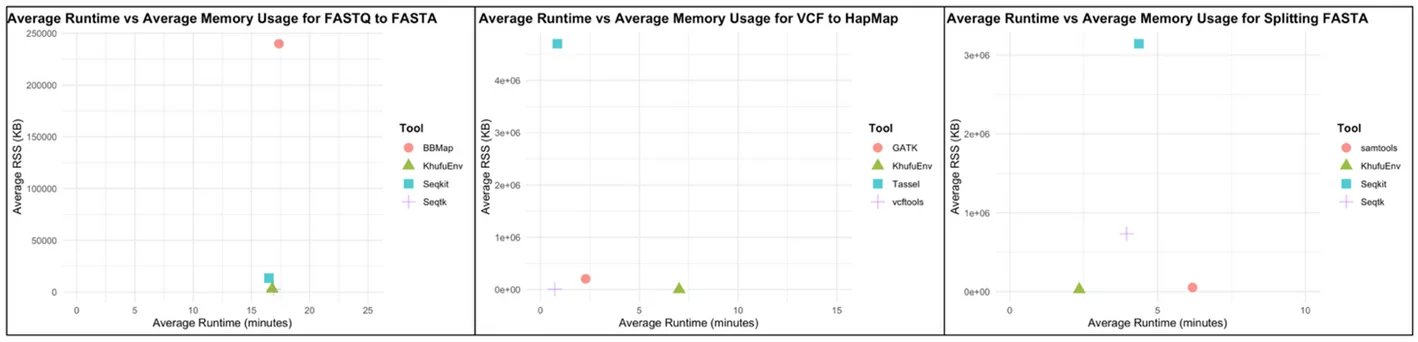
**A** Performance benchmarking of BBMap, KhufuEnv, Seqkit, and Seqtk tools for converting FASTQ to FASTA. **B** Performance benchmarking of GATK, KhufuEnv, Tassel, and VCFtools tools for converting VCF to HapMap. **C** Performance benchmarking of samtools, KhufuEnv, Seqtk, and Seqkit tools for splitting a FASTA file

Although the major advantage presented by the KhufuEnv is its breadth of tools for generating custom genomics pipelines, individual tools were compared to similar tools in other genomics tool kits for performance benchmarking. Common tasks were executed on three input file types, FASTQ, VCF, and FASTA, using publicly available datasets. Each kit’s command was run in parallel with the others and replicated five times. Since not all the tool kits tested had comparable functions for the example KhufuEnv function to benchmark, different tool kits were used with each comparison. For converting FASTQ to FASTA, the KhufuEnv tool was the second fastest tool after Seqkit and consumed less memory than BBMap and Seqkit (Fig. 11A and Supplementary File 6). While the KhufuEnv VCF to HapMap conversion tool took longer than GATK, Tassel [56], and VCFtools (Fig. 11B and Supplementary File 7), it required the least amount of memory to run. The KhufuEnv tool for splitting FASTA was the most computationally efficient tool tested, requiring the least amount of memory and time (Fig. 11C and Supplementary File 8).

Notably, in many of these comparisons, KhufuEnv tools were more flexible in requiring fewer associated input files compared to their counterparts. For instance, GATK [57] requires the generation of a sequence dictionary file (DICT) and a FASTA index file associated with the reference to convert VCF to HapMap, Tassel requires a sorted VCF file for conversion to HapMap, and samtools [68] and Seqtk require lists of chromosome name to loop through to extract chromosome headers for splitting (Supplementary Code 2). Time and resources to generate these additional files were not included in performance benchmarking.

## Discussion

More genomics data is being generated than ever before. This year, it is expected that 40 exabytes will be needed to store genomics data [2], equivalent to the data required to stream 1 billion hours (114,155 years) of standard definition content [58]. This behemoth of data being generated necessitates the development and implementation of tools for rapid data analysis, in addition to expanding the genomics data science workforce [59]. To address these challenges, we developed the KhufuEnv, a user-friendly, open-source auxiliary environment to assist analyzing genetics and genomics data. We have described some of the 132 tools present in its current release, along with the development of our custom HapMap and PanMap formats. While the standard HapMap has been a widely utilized file format for variant calls against linear genomes [20], to date, there has yet to be an equivalent format developed for visualizing long stretches of SVs. By numerically coding allele calls, long SVs and shorter mutations can be visualized side-by-side to facilitate ease for data wrangling within Unix or graphical user interface platforms.

In four case studies across animal and plant systems within autosome and allosome regions, we have benchmarked custom KhufuEnv pipelines to demonstrate the power and accuracy of its tools for trait mapping. Zhang et al. [26] identified a 4.18 Mb region (152,644,698–156,822,195) on chromosome 19 (B09) associated with cold tolerance during peanut germination, which was later fine-mapped to a 216 kb region (155,637,831–155,854,093) with Kompetitive Allele Specific PCR (KASP) markers. Beyond the variant calling, we accomplished finding a QTL near the fine-mapped region (155.2 Mb) (Fig. 4 and Supplementary File 2) using only nine lines of Unix code in a single environment, while the original authors required constructing a genetic map and QTL mapping with three softwares, SNPBinner [60], IciMapping [61], and BLAT [62], and two R packages, LinkageMapView [63] and R/qtl [64]. Similarly, in the case of identifying the SDR region in *Amborella*, the original authors utilized an extensive approach that incorporated TRIMMOMATIC [65] to generate k-mers for the WGS samples, identifying canonical 21-mers with Jellyfish [66], mapping all W-mers (female-specific k-mers) back to the haplotype assemblies with BWA-MEM, calculating coverage in sliding windows with BEDTools, and plotting with the R package karyoploteR [67]. We were able to accomplish identifying the SDR exclusively with KhufuEnv tools in seven lines of code beyond the initial pangenome graph construction and variant calling (Fig. 6A, B and Supplementary File 3).

Perhaps the greatest advantage of the KhufuEnv is its efficiency. In addition to exploiting tools to accurately de novo identify QTL which were previously characterized, even on a single thread, KhufuEnv tools identify such regions using a fraction of the time for identifying regions as listed in the previous studies (Table 1). De novo identification of the cold tolerance and SDR regions took less than five minutes and three minutes, respectively. Calculating FROH across different breeds was done in less than six minutes, and the process of de novo identifying the hairless canine QTL took less than one hour to run all subsets.

**Table 1:** Processing time for different KhufuEnv pipelines for benchmarking

Case study	References	Maximum Khufu processing time
Peanut cold tolerance	Zhang et al. [26]	4 min 0 s
*Amborella* SDR	Carey et al. [34]	2 min 56 s
Canine FROH	Meadows et al. [44]	2 min 20 s to 6 min 0 s
Canine hairlessness	Drögemüller et al. [52]	17 min 22 s to 40 min 2 s

Other advantages the KhufuEnv presents are its ability to read files from the standard input and write to standard output, allowing for more complex, pipeable actions to be done in a single line (with the exception of PanMap tools as PanMap tools output two files, a PanMap and a FASTA file of variant sequences). While Unix has several built-in tools that can be used for manipulating data files, some have limitations with regards to genomics data. For instance, “join” can be used to merge lines from two files based on a common field (Supplementary Figs. 4, 5). When using join to merge two HapMap files, the join tool only merges data based on the first column, “chr” (Supplementary Fig. 6). In the KhufuEnv, however, we can use the hapmapMerge tool to combine two HapMaps by joining two input HapMaps in the order they are passed or the hapmapConcatenate tool to combine row data for shared sample IDs to in two HapMaps (Supplementary Figs. 7 and 8, respectively). Although general statistics tools are useful for obtaining global views on genotyping data (and other datasets), Unix does not include built-in statistics tools. The KhufuEnvStats section can be extended to countless file types to easily extract statistics information.

While the KhufuEnv offers an integrative set of tools to manipulate and analyze genomics data, we acknowledge its limitations. Zhang et al. [26] developed a genetic map and exploited genetic linkage to identify QTL. Relying solely on phenotypic information and differences in allele frequencies between sample sets, we are not able to determine the phenotypic variation explained (PVE), ${R}^{2}$, or if the allele of interest behaves in an additive, dominant, or epistatic fashion. When utilizing our KhufuEnv approach for identifying a QTL for CED in canine (Fig. 10), it is relevant to note in this approach that several significant hits were found across the genome outside the region harboring *FOXI3* (Supplementary File 5). However, this is likely an artifact due to the small number of hairless dogs (11) we had to compare to a diverse group of over 1000 non-hairless dogs. The study by Drögemüller et al. [52] utilized data for 20 hairless and 19 coated Chinese Crested dogs for their genome-wide association study (GWAS). Having nearly double the number of hairless individuals than our study and closer related individuals without the trait of interest to compare with likely resolves the noise experienced in our benchmark. The hapmapGetROH tool in the current KhufuEnv release classifies ROH with a default stretch of 50 minimum bp of homozygosity. While this minimum can be user-customized, we have not yet incorporated a sliding window approach or customizations for allowable heterozygous sites within a window but anticipate improving this tool accordingly for the next release. Nonetheless, our results mimic the pattern demonstrated by Meadows et al. [44] (Fig. 8 and Supplementary File 4).

In its current state, the KhufuEnv prioritizes simple and quick processes which can be run on a single thread. We recognize that this prevents the integration of other tools such as DNA sequence aligners like BWA or variant callers like GATK into the environment itself and that users may have to rely on these programs during initial analyses if variant call files are not publicly available to them. Our needs for trait mapping inspired the present generation of KhufuEnv tools. The KhufuEnv currently does not have tools explicitly designed for manipulating GFF, BED, BAM, or SAM files (although some tools in our dataset processing section may be applicable) for which other suites, such as BEDTools and BBTools, support. As the KhufuEnv is an open-source resource, we will be integrating user feedback to design new tools dependent on community needs. Thus, we anticipate developing tools to specifically address other file types and data processing in future releases.

## Conclusions

We report the inaugural release of the KhufuEnv, an open-source toolbox to support genomic analyses. The KhufuEnv allows users to develop modular, custom pipelines for analyzing and manipulating genomics data in a standalone environment, and the custom HapMap and PanMap formats allow for visualizing variants called from linear assemblies and pangenomes, respectively, in a concise manner. As demonstrated by four case studies pertaining to plant and animal breeding, KhufuEnv tools can facilitate accurate and rapid de novo identification of relevant QTL for a variety of traits in diverse systems. While the example pipelines can be extended to other private and publicly available datasets for trait mapping, the 132 tools can be used to generate additional custom pipelines for analyzing virtually any variant call format described in the paper. Although its current state prioritizes speed, simplicity, and single-threaded tools, we look forward to incorporating additional tools and capabilities to suit user preferences in future KhufuEnv releases.

## Supplementary Information

Supplementary file 1. Detailed methods for the case study examples and benchmarking are provided.

Supplementary file 2. Raw ΔSNP-index values calculated by the KhufuEnv for case study 1.

Supplementary file 3. Raw allele frequency statistics calculated by the KhufuEnv for case study 2.

Supplementary file 4. FROH values calculated by the KhufuEnv tools for case study 3.

Supplementary file 5. Hits for the unique consensus hairless alleles compared to non-hairless samples generated by the KhufuEnv across entire genome for case study 4.

Supplementary file 6. Benchmarking average RSS and time for four tools to convert FASTQ to FASTA.

Supplementary file 7. Benchmarking average RSS and time for four tools to convert VCF to HapMap.

Supplementary file 8. Benchmarking average RSS and time for four tools to split FASTA.

Supplementary file 9. Figure S1. An example output of help information for a KhufuEnv tool. Figure S2. An example of the standard HapMap format. Figure S3. An example of the Khufu HapMap format. Figure S4. An example of the first HapMap to be merged with the hapmapMerge tool. Figure S5. An example of the second HapMap to be merged with the hapmapMerge tool. Figure S6. The output of using the Unix merge tool to attempt to combine information from the first and second HapMaps. FigureS7. The output of using the KhufuEnv hapmapMerge tool to combine information from the first and second HapMaps. Figure S8. The output of using the KhufuEnv hapmapConcatenate tool to combine row information for samples with the same identifier across two HapMaps.

Supplementary file 10. Supplementary code 1: As an example for the custom pipelines we have built with KhufuEnv tools, we have provided the hard code for the case studies.

Supplementary file 11. Supplementary code 2: The commands for conducting the performance benchmarking are provided for converting FASTQ to FASTA, VCF to HapMap, and FASTA splitting. See notes in the code file for additional commands needed to run some of the tools which were not considered as part of the average RSS and time.

## Data Availability

The KhufuEnv can be downloaded at [https://github.com/w-korani/KhufuEnv] (https://github.com/w-korani/KhufuEnv). All raw data used in this manuscript was derived from prior experiments associated with their respective citations. Input data which corresponds to the four case studies is available on FigShare: https://figshare.com/s/ec09ad1ba7906afac7f6. Additional testing datasets are available within the KhufuEnv.

## Abbreviations

CED: Canine ectodermal dysplasia
CS: Cold-susceptible
CT: Cold-tolerant
FROH: Fraction of runs of homozygosity
GFA: Graphical fragment assembly
GWAS: Genome-wide association study
KASP: Kompetitive allele specific PCR
MAF: Minor allele frequency
NCBI: National Center for Biotechnology Information
PVE: Percent variance explained
QTL: Quantitative trait locus
RIL: Recombinant inbred line
ROH: Runs of homozygosity
SDR: Sex-determination region
SNP: Single nucleotide polymorphism
SRA: Sequence Read Archive
SV: Structural variant
VCF: Variant call format
WGS: Whole-genome sequencing
