# Supplementary material for: KhufuEnv, an auxiliary toolkit for building computational pipelines for plant and animal breeding
Source: BMC Bioinformatics. 2026 Jun 3;27:198. doi: 10.1186/s12859-026-06499-9 (PMC13508277; doi:10.1186/s12859-026-06499-9)
Supplement: Supplementary file 1 [file 12859_2026_6499_MOESM1_ESM.docx]

**Supplementary File 1: Detailed Methods**

To demonstrate the flexibility of the KhufuEnv, we selected publicly available datasets for three distinct biological systems as described in the above case studies: the allotetraploid peanut, *Amborella* *trichopoda*, and canine. All case studies were run in the HudsonAlpha Institute for Biotechnology high performance computer (HPC) environment on a Rocky Linux operating system using the tools in the order described under the “Results” section. Each task ran on less than 2.7 GB of memory, and total running time for each study is listed in Table 1. While not necessary for implementation tools in the KhufuEnv, result data extracted from KhufuEnv tools were visualized using the ggplot2 package [1] in R. Raw ΔSNP-index values in case study 1 were plotted in Fig. 4. Allele frequencies which differed by > 0.75 between male and female samples in case study 2 were transformed to “1.0”, while allele frequencies which differed by ≤ 0.75 were transformed to “0” and plotted in Fig. 6A and Fig. 6B to designate categorial hits for sex-determination regions (SDRs). Boxplots in Fig. 8 were generated from raw fraction of runs of homozygosity $F_{ROH}$ calculated using KhufuEnv tools for each of the three breeds tested in case study 3. Positions for which consensus hairless alleles were unique compared to all individual non-hairless samples were plotted categorically in Fig. 10 with “1.0” designating a unique hit and “0” representing a non-unique region in chromosome 17 for case study 4.

KhufuEnv tools were compared to tools in other genomics toolkits for comparing resource usage (average RSS) and time. Conversion of a FASTQ to FASTA was tested using the KhufuEnv, BBMap (version 38.42), Seqkit (version 2.10.0), and Seqtk (version 1.4) using the fastq2fasta, reformat.sh, fq2fa, and seq -a commands, respectively (Supplementary Code 2). The input FASTQ was a 31.4Gb PacBio HiFi long-read file of a peanut Multi-Parent Advanced Generation Inter-Cross (MAGIC) population founder (<https://www.ncbi.nlm.nih.gov/sra/?term=SRR32237154>) [2]). Conversion of VCF to HapMap was tested using the KhufuEnv, GATK (version 4.6), Tassel (version 5.0), and vcftools (version 0.1.16) tools. PLINK BED/BIM/FAM variant files for 1,122 cats using a 340k single nucleotide polymorphism mapping array [3] (https://datadryad.org/dataset/doi:10.5061/dryad.f1vhhmgwp) were converted into a VCF file with the plink command *plink --bfile Hernandez_Hayward_feline3 --recode vcf --allow-extra-chr --out Hernandez_Hayward_feline3*. To make the VCF file compatible with Tassel, the Tassel SortGenotypeFilePlugin was utilized to sort the VCF by chromosome name, and the sorted VCF was utilized as input for all four tools. The GATK tool required the generation of an index for the Abyssinian domestic cat genome assembly (Felis_catus-6.2) (<https://www.ncbi.nlm.nih.gov/datasets/genome/GCA_000181335.2/>), in addition to a DICT file (Supplementary Code 2). FASTA splitting was done using the KhufuEnv, samtools (version 1.19.2), Seqtk, and Seqkit tools. The input FASTA file was the same domestic cat genome assembly (Felis_catus-6.2), containing 5,500 scaffolds to be split. Ten GB of memory were allocated for each performance benchmarking script. Each script was run in a batch script in parallel with the others for five replications each, and the averages for time and average RSS (Supplementary Files 5-7) were extracted with the command: *sacct -j [job ID] --format=JobID,Elapsed,AveRSS*. The average values of time and average RSS per tool were plotted using the ggplot2 package [1] in R.

**References**

1.	Wickham H: **ggplot2: Elegant graphics for data analysis**, 2 edn; 2016.

2.	Lee K, Korani W, Bentz PC, Pokhrel S, Ozias-Akins P, Harkess A, Vaughn J, Clevenger J: **Long-Read Low-Pass Sequencing for High-Resolution Trait Mapping**. *bioRxiv* 2025:2025.2001.2009.632261.

3.	Hernandez I, Hayward JJ, Brockman JA, White ME, Mouttham L, Wilcox EA, Garrison S, Castelhano MG, Loftus JP, Gomes FE *et al*: **Complex Feline Disease Mapping Using a Dense Genotyping Array**. *Frontiers in Veterinary Science* 2022, **Volume 9 - 2022**.
