# Supplementary material for: KhufuEnv, an auxiliary toolkit for building computational pipelines for plant and animal breeding
Source: BMC Bioinformatics. 2026 Jun 3;27:198. doi: 10.1186/s12859-026-06499-9 (PMC13508277; doi:10.1186/s12859-026-06499-9)
Supplement: Supplementary file 2 [file 12859_2026_6499_MOESM9_ESM.docx]

**Additional File 9: Supplementary Figures**

Supplementary Figure 2: An example of the standard HapMap file format developed by The International HapMap Consortium (2003). This is an example output from running the command “**cat test1.stdhapmap | column -t**”.

Supplementary Figure 1: The KhufuEnvHelp [tool] command outputs information regarding a tool’s description, parameters, and practice files for using the tool. This is an example output from running the command “**KhufuEnvHelp vcf2hapmap**”.

Supplementary Figure 3: An example of the condense Khufu HapMap format. This is an example output from converting the test1.stdhapmap to a Khufu HapMap running the command “**stdhapmap2hapmap test1.stdhapmap > test1.std.toHAPMAP**” then “**cat test1.std.toHAPMAP | column -t**”.

Supplementary Figure 4: An example HapMap displayed by running the command “**cat test1.hapmap | column -t**”.

Supplementary Figure 6: Instead of combining the genotyping data for each of the samples from the HapMaps in Supplementary Figs. 4 and 5, the join function in Unix only merges data based on the first column when using the commands “**join test1.hapmap test2.hapmap > merged.hapmap**” and “**cat merged.hapmap | column -t | head**”.

Supplementary Figure 5: An example HapMap displayed by running the command “**cat test2.hapmap | column -t**”.

Supplementary Figure 8: The KhufuEnv hapmapConcatenate tool combines row data for samples of shared ID’s in two input HapMaps. Commands used to generate the merged HapMap were “**hapmapConcatenate test1.hapmap test2.hapmap > Concat.hapmap**” and “**cat Concat.hapmap | column -t**”.

Supplementary Figure 7: Alternative to the Unix join function, the KhufuEnv hapmapMerge tool combines information from two HapMaps such that row data existing in the right (secondary) HapMap that does not exist in the left HapMap (primary) will be dropped. Commands used to generate the merged HapMap were “**hapmapMerge test1.hapmap test2.hapmap > Merge.hapmap**” and “**cat Merge.hapmap | column -t**”.
